# Ca^2+^ regulation of constitutive vesicle trafficking

**DOI:** 10.12703/r/11-6

**Published:** 2022-03-09

**Authors:** John Sargeant, Jesse C Hay

**Affiliations:** 1 Division of Biological Sciences & Center for Structural & Functional Neuroscience, University of Montana, 32 Campus Drive, Missoula, MT 59812, USA

**Keywords:** vesicle trafficking, calcium, secretion, Golgi, endoplasmic reticulum, lysosomes, late endosomes, calcium channel, calcium signaling, vesicle coat, apoptosis-linked gene 2 (ALG-2)

## Abstract

Constitutive vesicle trafficking is the default pathway used by all cells for movement of intracellular cargoes between subcellular compartments and in and out of the cell. Classically, constitutive trafficking was thought to be continuous and unregulated, in contrast to regulated secretion, wherein vesicles are stored intracellularly until undergoing synchronous membrane fusion following a Ca^2+^ signal. However, as shown in the literature reviewed here, many continuous trafficking steps can be up- or down-regulated by Ca^2+^, including several steps associated with human pathologies. Notably, we describe a series of Ca^2+^ pumps, channels, Ca^2+^-binding effector proteins, and their trafficking machinery targets that together regulate the flux of cargo in response to genetic alterations as well as baseline and agonist-dependent Ca^2+^ signals. Here, we review the most recent advances, organized by organellar location, that establish the importance of these components in trafficking steps. Ultimately, we conclude that Ca^2+^ regulates an expanding series of distinct mechanistic steps. Furthermore, the involvement of Ca^2+^ in trafficking is complex. For example, in some cases, the same Ca^2+^ effectors regulate surprisingly distinct trafficking steps, or even the same trafficking step with opposing influences, through binding to different target proteins.

## Introduction

Eukaryotic cells are in a constant state of membrane maintenance, renewal, and expansion, accomplished by an interwoven web of constitutive vesicle trafficking pathways connecting the endoplasmic reticulum (ER), Golgi, plasma membrane (PM), and the endolysosomal system. Classically, constitutive secretion is thought to be continuous and unregulated. This contrasts with the highly controlled, regulated secretory pathway in which proteins stored in intracellular granules are released en masse in response to external factors or signals. However, constitutive secretion is also regulated. By way of example, during mitosis in mammalian cells, the Golgi is fragmented for partitioning into progeny cells. This transition from S-to-M phase necessitates that most, if not all, membrane trafficking pathways are temporarily shut down, only to be resumed later during cytokinesis^1^. Alternatively, the differentiation of B cells to plasma cells requires a marked increase in secretory capacity, driven in part by sequential waves of protein expression^2^. However, there is still much to learn about the regulation of constitutive secretion. The milieu of ions, lipids, and proteins that constitutive trafficking externalizes, degrades, or delivers to various organelles affects nearly every aspect of cell metabolism, making dynamic regulation of these pathways of great significance to biomedical research.

**Figure 1.**
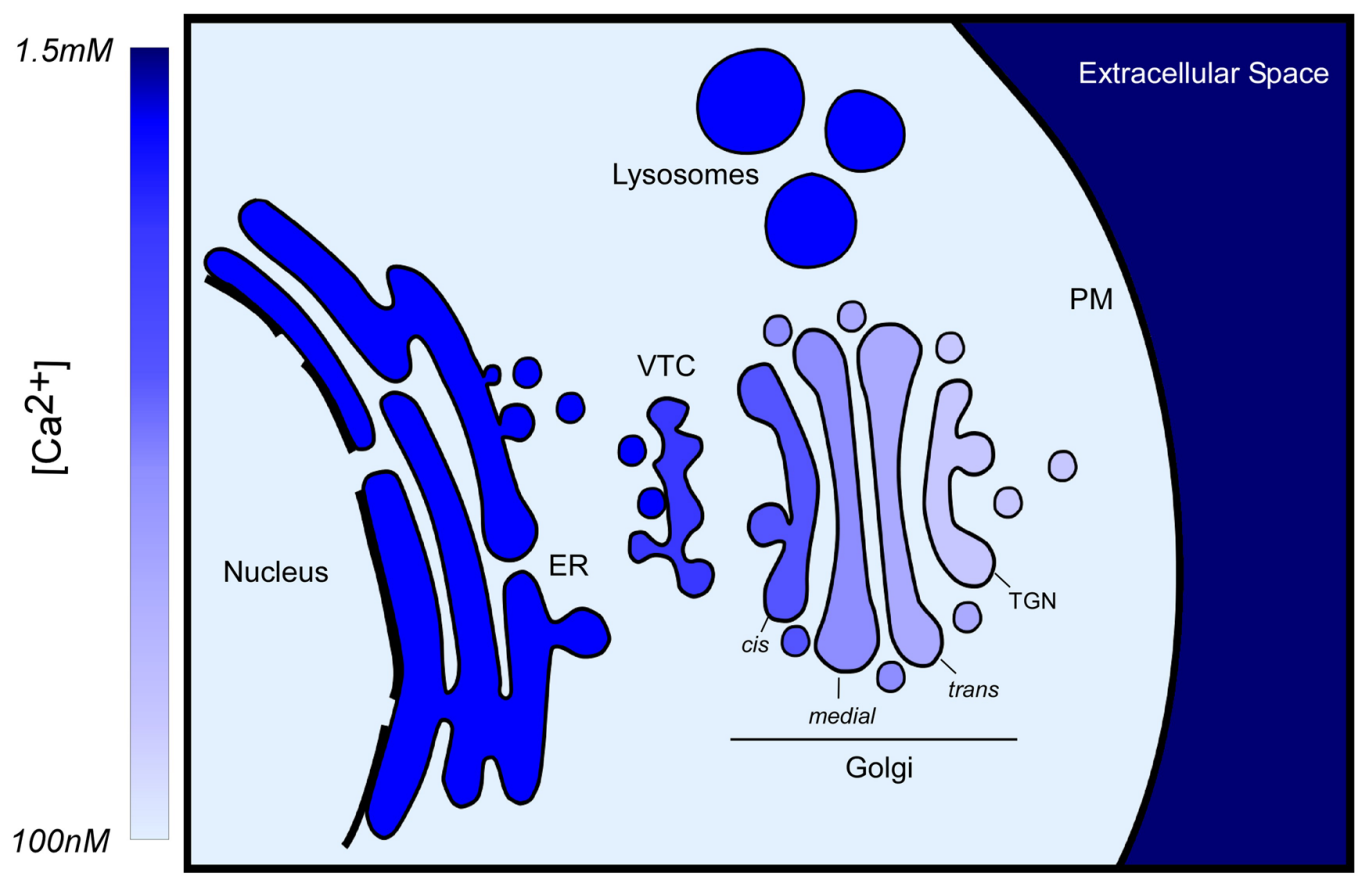
Ca^2+^ concentrations in constitutive trafficking. Of the Ca^2+^ pools depicted, the low end is the cytosol, with a free [Ca^2+^] of about 100 nM. The concentration of Ca^2+^ decreases from the endoplasmic reticulum (ER) (~500 µM) to the cis*-*Golgi (~300 µM) to the trans-Golgi network (TGN) (50–100 µM)^8,9^, in the same direction as anterograde secretion. Lysosomes contain a similar [Ca^2+^] to the ER^8,10^. At the high end is the extracellular space with typical [Ca^2+^] of 1.2 to 1.5 mM^8^. PM, plasma membrane; VTC, vesicular tubular cluster.

Intracellular Ca^2+^ is actively sequestered into several organelles for use in a myriad of signaling processes^3^. Many of these Ca^2+^ stores, such as the ER, Golgi, and lysosome, are prominent in constitutive vesicle trafficking pathways (Figure 1). Interestingly, the concentration of luminal Ca^2+^ decreases from the ER to the PM, the same direction as the flow of cargo, suggesting a role for Ca^2+^ in directing cargo progression or sorting. Ca^2+^ is well known for its role in activation of regulated exocytosis. However, Ca^2+^ has also been suggested to play a role as a regulator of constitutive secretion. An often-cited study showed that vesicular tubular cluster (VTC)-to-Golgi and Golgi-to-ER retrograde trafficking is inhibited by the fast Ca^2+^ chelator 1,2-bis(2-aminophenoxy)ethane-N,N,N′,N′-tetraacetic acid (BAPTA) but not the slower chelator of comparable Ca^2+^ affinity, ethylene glycol-bis(2-aminoethylether)-N,N,N′,N′-tetraacetic acid (EGTA)^4^. In addition to illustrating a role for calcium in constitutive secretion, that study implied tight spatial coupling of the vesicular fusion machinery and Ca^2+^, wherein the putative fusion site is close to the Ca^2+^ release site. Since then, further evidence continued to mount and various groups have implicated Ca^2+^ in the fusion of yeast vacuoles^5^, the heterotypic fusion of late endosomes with lysosomes, or the reformation of lysosomes from hybrid compartments^6^.

**Figure 2.**
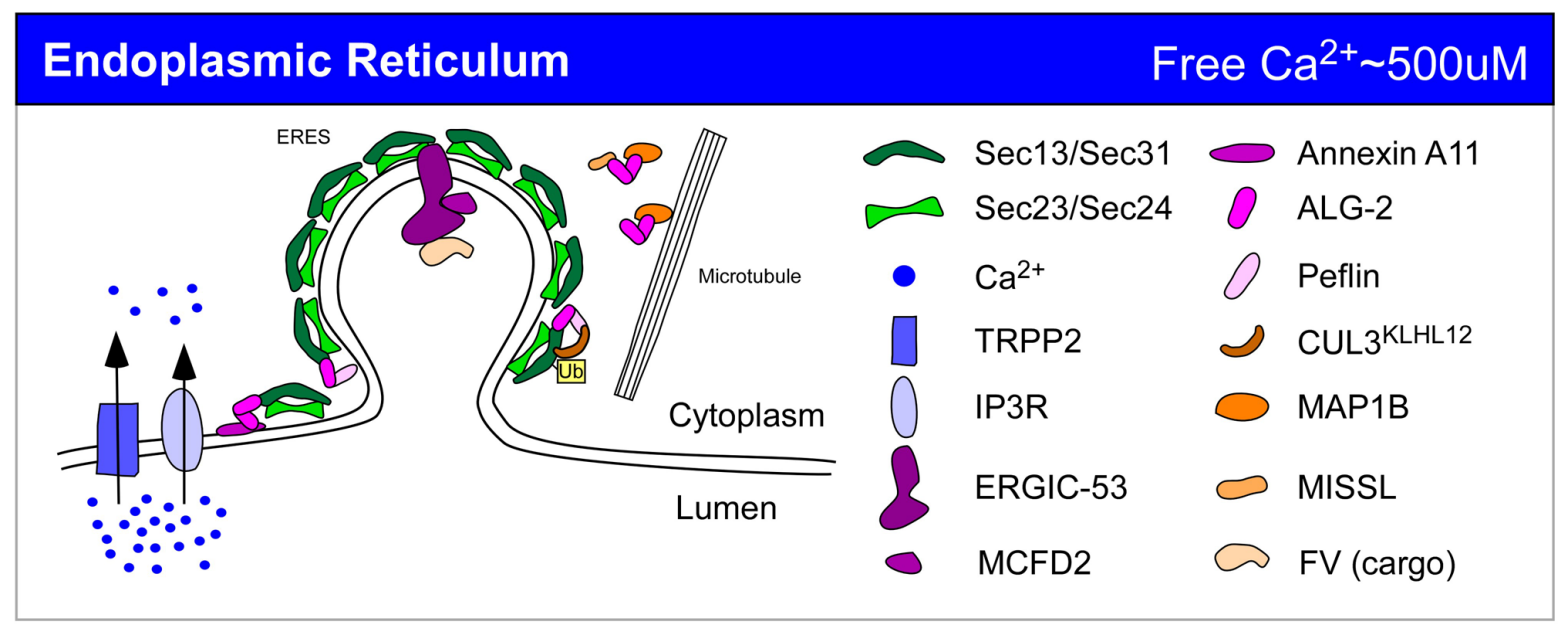
Ca^2+^ regulation of constitutive secretion at the endoplasmic reticulum. Prominent examples from the text are depicted and color-coded at an example endoplasmic reticulum exit site (ERES). Greens: trafficking machinery; pinks/purples: Ca^2+^-binding proteins; blues: Ca^2+^ pumps or channels; oranges: accessory proteins or cargo. Ub, monoubiquitination.

**Figure 3.**
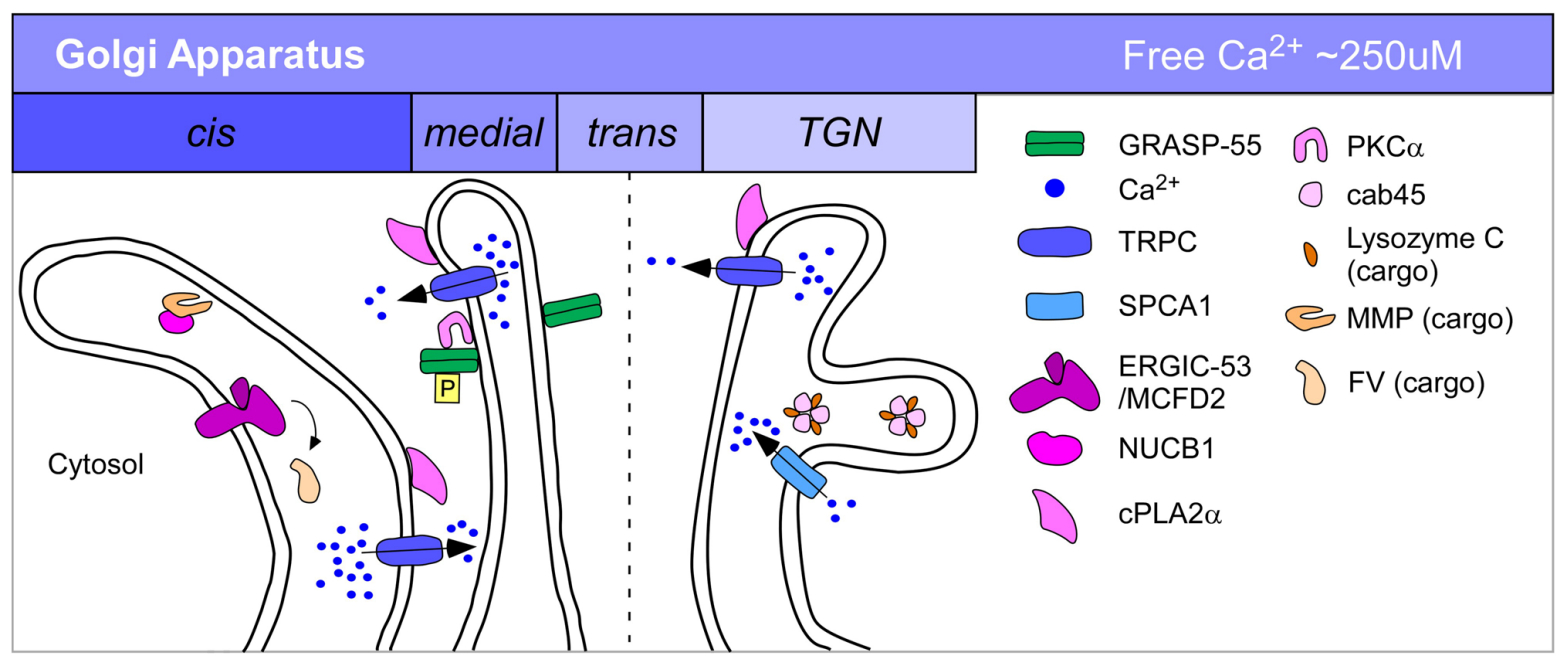
Ca^2+^ regulation of constitutive secretion through the Golgi and trans-Golgi network (TGN). Prominent examples from the text are depicted and color-coded in various Golgi compartments. Greens: trafficking machinery; pinks/purples: Ca^2+^-binding proteins; blues: Ca^2+^ pumps or channels; oranges: accessory proteins or cargo. P, phosphorylation.

**Figure 4.**
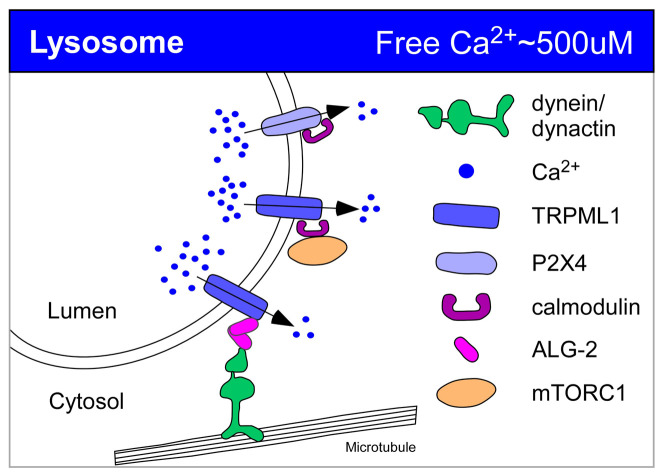
Ca^2+^ regulation of constitutive secretion at the lysosome. Prominent examples from the text are depicted and color-coded. Greens: trafficking machinery; pinks/purples: Ca^2+^-binding proteins; blues: Ca^2+^ pumps or channels; oranges: accessory proteins or cargo.

In this review, we discuss multiple proteins that appear to regulate constitutive trafficking and have direct or indirect ties to Ca^2+^, focusing mainly on studies after 2007, when we published a review on the same topic^7^. We discuss a variety of secretory effectors, organized by the organelle wherein the primary secretory effects spatially occur, that modulate secretion via their capacity to bind Ca^2+^. In addition to these Ca^2+^ effectors, what has been most obvious in recent years is the rise in the number of calcium pumps or channels that have been found to affect constitutive trafficking. Channels mobilize Ca^2+^ and create a dynamic Ca^2+^ landscape in the cytoplasm^8^ but still must act upstream of Ca^2+^ sensors and trafficking machinery effectors to influence trafficking. Pumps, on the other hand, tend to affect trafficking on the luminal side by providing sufficiently high Ca^2+^ environments for Ca^2+^-dependent sorting or retention processes. Prominent example studies from the text are briefly summarized in Table 1 and Figure 2, Figure 3, and Figure 4. Some of the examples that we discuss, such as certain TRP channel family members of non-selective ion channels, have been implicated in secretion changes yet so far have not been matched up with Ca^2+^ sensors that could translate a Ca^2+^ flux into changes in secretion. This may mean that the critical Ca^2+^-binding effectors or channel-binding partners have not been defined yet.

**Table 1.** Summary of Ca^2+^ regulation of vesicle trafficking organized by organelle and order of appearance in the text.

Endoplasmic reticulum					
Primary reference(s), by first author	Ca^2+^ channel/ pump	Ca^2+^-binding effector	Links to Ca^2+^ flux/homeostasis	Links to trafficking machinery	Observed trafficking effects
**Le Corre (2014)^16^;** **Sammels (2010)^19^**	TRPP2	-	TRPP2 KD increased releasable Ca^2+^	TRPP2 KD upregulated COPII expression	TRPP2 KD increased collagen secretion
**La Cour (2013)^35^**	-	ALG-2	Ca^2+^-bound ALG-2 binds Sec31	Ca^2+^-bound ALG-2 potentiated binding of Sec31 to Sec23	Ca^2+^-bound ALG-2 inhibited COPII vesicle budding
**Shibata (2015)^46^**	-	ALG-2/AnxA11	ALG-2 binds AnxA11 Ca^2+^- dependently	ALG-2 couples AnxA11 to Sec31A	AnxA11 or ALG-2 KD increased ER-to- Golgi transport of VSV-G
**Takahara (2017)^48^**	-	ALG-2/MISSL/ MAP1B	MISSL colocalizes with ALG-2 in response Ca^2+^	MISSL-ALG-2-MAP1B may sequester ALG-2	KD of MISSL or ALG-2 decreased SEAP secretion
**McGourty (2016)^40^**	-	ALG-2/peflin /CUL3^KLHL12^	Ca^2+^-dependent assoc. of KLHL12 with Sec31	CUL3^KLHL12^ monoubiquitinated sec31A	Ubiq. complex required for collagen I secretion
**Sargeant (2021)^39^**	IP3R	ALG-2/peflin	Pulse of Ca^2+^ signaling	Increased Sec31 targeting to ERES	Increased ER-to-Golgi transport
**Sargeant (cont.)^39^**	IP3R	ALG-2/peflin	Continuous Ca^2+^ signaling	Decreased Sec31 targeting to ERES	Decreased ER-to Golgi transport
**Held (2021)^21^**	IP3R	ALG-2/peflin	IP3R-3 KD increased Ca^2+^ signaling	IP3R-3 KD increased ALG-2 and COPII coat at ERES	IP3R-3 KD increased ER-to-Golgi transport
**Cho (2018)^51^;** **(2020)^50^**	-	-	24-hour extremes of low or high Ca^2+^	Sec31 S694 O-GlcNACylated/de- acylated, respect.	Golgi structure modulated by Sec31 acylation
**Trychta (2018)^55^**	-	-	24-hour TG, depleted luminal Ca^2+^	Overwhelmed and upregulated KDEL receptors	Secretion of ER-resident proteins
**Zheng (2013)^66^**	-	ERGIC-53/ LMAN-3	Ca^2+^-dependent binding/release of luminal cargo	ERGIC-53/LMAN-3 is a COPII client membrane protein	Req. for trafficking of coag. factors, neurorec. and others
Golgi apparatus					
Primary reference(s), by first author	Ca^2+^ channel/ pump	Ca^2+^-binding effector	Links to Ca^2+^ flux/homeostasis	Links to trafficking machinery	Observed trafficking effects
**Lavender (2008)^82^**	TRPC3/TRPC7	-	Presumed change of steady-state Ca^2+^	-	TRPC3,7 OE increased SEAP secretion 2x-4x
**Ireland (2020)^80^**	-	PKCα	Ca^2+^ agonist or TG for up to 2 hours	PKCα-mediated phosphorylation of GRASP55	Golgi fragmentation and increased intra- Golgi transport
**San Pietro** **(2009)^88^**	-	cPLA_2_α	Presumed cargo-dependent Ca^2+^ release at Golgi	cPLA_2_α KD or inhibition reduced inter- cisternal tubules	cPLA_2_α KD inhibited intra-Golgi transport of VSV-G
**Regan-Klapisz** **(2009)^91^**	-	cPLA_2_α	Presumed Ca^2+^ release from Golgi	cPLA_2_α KD changed Golgi morphology	KD accumulated junctional proteins in the Golgi
**Mukherjee** **(2016)^78^**	pmr1 (SPCA1 homolog)	Possible direct effect of Ca^2+^	sly41 OE increased cytoplasmic Ca^2+^	Bypassed lack of p115 homolog Uso1	Increased fusion of COPII vesicles with Golgi
**Grice (2010)^96^**	SPCA1	-	SPCA1 KD decreased Golgi luminal Ca^2+^	-	Blocked IGF1R trafficking/ maturation at the TGN
**Deng (2018)^97^;** **von Blume (2012)^98^**	SPCA1	cab45	SPCA1 KD decreased Golgi luminal Ca^2+^	cab45 oligomerized Ca^2+^-dependently with select cargoes in TGN	SPCA1 or cab45 KD blocked sorting of lysozyme C in TGN
**Larkin (2016)^103^;** **Brodeur (2009)^100^**	-	NUCB1	-	NUCB1 req. for rab7-dep. recruitment of retromer coat to LEs	NUCB1 KD caused lysosomal accum. of Mann-6P receptors
**Pacheco-** **Fernandez (2020)^106^**	-	NUCB1	NUCB1 KO reduces cis Golgi luminal Ca^2+^	NUCB1 directly bound cargo MMPs in cis-Golgi lumen	NUCB1 KO delays intra-Golgi transport of MMPs
Endosome/Lysosome					
Primary reference(s), by first author	Ca^2+^ channel/ pump	Ca^2+^-binding effector	Links to Ca^2+^ flux/homeostasis	Links to trafficking machinery	Observed trafficking effects
**Yang (2019)^115^;** **Cao (2017)^113^;** **Dong(2010)^114^**	TRPML1	CaM	TRPML1 OE enhanced LEL Ca^2+^ release	CaM recruited mTORC1 to LEL	TRMPL1 KD produced enlarged LEL
**Li (2019)^112^**	TRPML1	-	TRPML1 Ca^2+^ release regulated by ceramidase	TRPML1 inhibition blocked lysosome- MVB interactions	TRPML1 inhibition stimulated exosome release
**Li (2016)^10^**	TRPML1	ALG-2/dynein- dynactin	TRPML1 Ca^2+^ release regulated by PI(3,5)P_2_	Ca^2+^ recruited ALG-2 and dynein/ dynactin to TRPML1	Permits perinuclear positioning of LEL
**Cao (2015)^117^**	P2X4	CaM	P2X4 released Ca^2+^ from the LEL pH-dependently	CaM presumed to activate fusion machinery	P2X4 OE promotes endolysosome fusion

CaM, calmodulin; ER, endoplasmic reticulum; ERES, endoplasmic reticulum exit site; KD, knockdown; KO, knockout; LE, late endosome; LEL, late endosome/lysosome; MMP, matrix metalloproteinase; MVB, multivesicular body; OE, over-expression; SEAP, secretory alkaline phosphatase; TG, trans-Golgi; TGN, trans-Golgi network.

## Calcium and secretion

### Endoplasmic reticulum

The ER folds and assembles newly synthesized proteins destined for trafficking via the secretory pathway, dysregulation of which leads to the unfolded protein response (UPR), reviewed elsewhere^11,12^ but not covered in this review except regarding Ca^2+^-specific observations. In addition, the ER acts as a principal Ca^2+^ store within cells facilitating Ca^2+^ signals in the cytosol or multiple organelles via ER-organelle contact sites^8,13–15^. In this section, we describe effects on secretion via ER-localized Ca^2+^ channels or their cytosolic effectors; finally, we note ways in which luminal calcium has been described to affect secretion.

Originally studied for its role in polycystic kidney disease, TRPP2 was suggested to have a role in altered extracellular matrix (ECM) integrity. TRPP2 localizes to the ER membrane, although it is also present at the PM and primary cilia^16^. TRPP2, like all TRP family members, functions as a cation channel, principally directing the flow of Ca^2+^ into the cytosol. TRPP2 knockdown (KD) caused enhanced production of type II collagen in the zebrafish notochord sheath^16^, a trait reversed by expression of an ER-retained mutant of TRPP2^17^. This implied that TRPP2 activity in the ER could directly or indirectly affect collagen translation or vesicle trafficking. Indeed, TRPP2 depletion led to an increase in the mRNAs of inner COPII coat components Sec23B, Sec24C, and Sec24D^16^, factors predicted to increase rates of ER-to-Golgi trafficking. Given that the increased coat expression could also be a compensation mechanism for loss of TRPP2, it is unclear whether TRPP2 depletion itself increased secretion. However, COPII coat protein KD or chemical inhibition of ER-to-Golgi transport via low-level brefeldin A (BFA) treatment was sufficient to reverse the enhanced collagen and aberrant morphology in the notochord sheath. This implies that the secretory pathway was an important target for manifesting the ECM abnormalities. Ultimately, in that study, a specific effector mechanism was not implicated nor were Ca^2+^ dynamics monitored, but previous work had shown that TRPP2 significantly decreased ER Ca^2+^^18^ and increased ER agonist-stimulated release of Ca^2+^^19^ or reduced it in the case of mutant TRPP2^20^. These findings are reminiscent of a study^21^ focusing on a separate Ca^2+^ channel: inositol trisphosphate receptor-3 (IP3R-3). IP3Rs are broadly localized; they are present at the Golgi^22^), nucleus^23–25^, and the PM^26,27^ but are by far most abundant at the cells’ largest Ca^2+^ store: the ER^28^. Held *et al*. showed that IP3R-3 depletion increased spontaneous Ca^2+^ oscillations and produced an increase in ER-to-Golgi trafficking of a VSV-G transmembrane cargo^21^. It is possible that the two channels—IP3R and TRPP2—regulate trafficking via the same mechanism, possibly mediated in part by the demonstrated ability of TRPP2 to bind to ER IP3R and potentiate its activity^19^.

Changes in local Ca^2+^ concentrations, mediated by changes in channel expression (as described above) or signaling events, likely affect secretion via direct binding of Ca^2+^ to effectors. At the ER, no effectors are more prominent than the cytosolic penta-EF-hand (PEF) proteins. Common features of this family include (1) five serial Ca^2+^-binding EF-hand motifs, (2) capacity to dimerize via EF-hand domains, and (3) Ca^2+^-dependent effector binding, often at membrane sites. Of this family, the most studied is apoptosis-linked gene-2 (ALG-2). ALG-2 has more than 20 Ca^2+^-dependent-binding partners and possesses several binding pockets for effectors, implying that one ALG-2 homodimer can bind two or more target proteins simultaneously in response to Ca^2+^-induced conformational changes^29^. Furthermore, in mammalian cells, ALG-2 is present in two splice forms that have a different spectrum of interaction partners^30^. Early research on ALG-2 in secretion indicated that it localizes principally with Sec31 at ER exit sites (ERES) in a manner concomitant with intracellular calcium oscillations^31–33^, suggesting a role for ALG-2 in affecting ERES function in response to a Ca^2+^ signal. Other early work reported that recombinant ALG-2 inhibited COPII vesicle budding and/or fusion *in vitro*^34,35^, implying an inhibitory role for ALG-2. Other cellular studies showed that recombinant ALG-2 bound to Sec31 was able to stabilize ERES^31,36^, and dominant-negative ALG-2^37^ and depletion^38^ studies also indicated a positive regulatory role. Recently, our lab helped reconcile some of these apparent contradictions by demonstrating that ALG-2 can either stimulate or inhibit ER-to-Golgi transport in the same system^39^. This duality in the regulatory role of ALG-2 may come down to multiple partners it can bind under different circumstances. In this review, we will discuss multiple ALG-2 protein complexes that can or do affect secretion.

In the basal state, in the absence of significant cytosolic Ca^2+^ oscillations, ALG-2 binds to the outer COPII coat subunit Sec31^31,32^, where it colocalizes with another PEF protein: peflin^39,40^. Likely, the colocalized peflin and ALG-2 at ERES are comprised of the earlier reported 1:1 heterodimer, observed *in vitro* to dissociate in response to Ca^2+^^41^. Interestingly, depletion of peflin in NRK cells produced increased ALG-2 at ERES^38^ and an increase in ER-to-Golgi trafficking of multiple COPII cargoes^39^, a situation reminiscent of ALG-2-binding to ERES in response to intracellular Ca^2+^ oscillations^33^, indicating a straightforward positive and negative role for ALG-2 and peflin at ERES, respectively. Furthermore, double KD of ALG-2 and peflin did not decrease transport below control levels, indicating that neither is actually required for secretion and both are purely regulatory in this context. Importantly, this result is in partial contrast to that of another study. McGourty *et al*. showed that the ubiquitin ligase CUL3 and its adaptor KLHL12 required both peflin and ALG-2 together for recognition and ubiquitination of Sec31 and further that both PEF proteins were positively required for collagen I secretion^40^. In that study, treatment of cells with histamine or ionomycin caused enlargement of COPII membrane sites or vesicles in an ALG-2- and peflin-dependent manner, suggesting activation of collagen export in response to surges in cytoplasmic Ca^2+^. The reason for the opposite effect of peflin depletion in Sargeant *et al*.^39^ and McGourty *et al*.^40^ is not known.

Other binding partners for ALG-2—for example, annexin A11 (Anxa11)—continue to support a regulatory role for ALG-2. The role of the annexin family of Ca^2+^- and lipid-binding proteins in trafficking is long and complex; a 2017 review discussed the potential involvement of annexins in a variety of plant membrane trafficking steps^42^. Briefly, there is very strong evidence that annexin A2 is involved in regulated exocytosis of chromaffin granules^43^ as well as several annexins being involved in Ca^2+^-dependent PM repair^44,45^, but the role of Anxa11 in ER-to-Golgi transport seems to be a unique example of an annexin playing a regulatory role at a constitutive trafficking step. At ERES, Anxa11, like ALG-2, has a role in stabilizing Sec31A^46^. There, Anxa11 likely anchors the ALG-2-Sec31A complex directly to the phospholipid bilayer, stabilizing the Sec31A interaction with ALG-2 rather than competing with it. Additionally, studies testing specific mutants that disrupt ALG-2-Anxa11-binding but not Sec31A-binding suggest that one ALG-2 molecule can bind Sec31A and Anxa11 at the same time, potentially through different binding sites. If this is true, then Anxa11 would not exactly be an additional ALG-2 target but rather an ALG-2 “co-effector” of Ca^2+^ since Anxa11 is itself a Ca^2+^-binding protein whose interactions with phospholipids could be independently modulated by Ca^2+^, separately from the Ca^2+^ modulation of ALG-2-binding to Anxa11 and Sec31A. This dual Ca^2+^-sensing unit could create complex functional responses, as has been observed in the Ca^2+^-dependent changes of ALG-2 in ER-to-Golgi transport. Also in support of more complex functions of ALG-2, ALG-2 has been reported to bind trk-fused gene (TFG) Ca^2+^-dependently at ERES in a Sec31A-independent manner^47^. GFP-MAPK1-interacting and spindle-stabilizing like (GFP-MISSL) also colocalized with ALG-2 in response to increased Ca^2+^^48^. The effector complex responsible for this targeting phenomenon appeared to be distinct from the Sec31A-ALG-2-Anxa11 or ALG-2-TFG complexes and was comprised of ALG-2 bound to both MISSL and MAP1B (microtubule-associated protein 1B) but not Sec31A. MISSL itself appeared to have a positive role in secretion as its KD attenuated secretion of SEAP (secretory alkaline phosphatase), possibly via sequestration of MAP1B, KD of which reversed the secretion defect. Furthermore, since the MISSL-ALG-2-MAP1B effector complex does not contain a member of the transport machinery, its effects on transport could be indirect (for example, by buffering ALG-2 availability for the Anxa11-ALG-2-Sec31A complex). Collectively, these studies indicate that ALG-2 has the capacity to serve multiple roles in secretion through binding to an array of target proteins.

Recently, our lab showed that distinct calcium signals and patterns can cause opposite effects on ER export in an ALG-2-dependent manner^39^. In NRK cells, exposure to histamine or ATP for up to 2.5 hours caused an ALG-2-dependent depression of ER-to-Golgi trafficking, but in neuroendocrine PC12 cells, the same treatment caused ALG-2-dependent enhancement of transport. Another recent report found a stimulatory effect of ER Ca^2+^ release in the ER-to-Golgi transport of a bulk flow cargo in HeLa cells^49^. We noted that the important distinction between a stimulatory or inhibitory effect of Ca^2+^ is not the cell type per se but the pattern and longevity of the Ca^2+^ signal produced, since by adjusting the pattern of signaling using different protocols, it was found that NRK cells could *either* down-regulate *or* up-regulate ER export in response to Ca^2+^ signaling in an ALG-2-dependent manner^39^. Mechanistically, the Ca^2+^-dependent decrease of ER export co-occurred with less COPII outer shell and more peflin at ERES but the opposite was true for the Ca^2+^-dependent enhancement of ER export. We proposed that different Ca^2+^ signals were able to elicit different effects via ALG-2, likely via other ALG-2- binding partners that may bind in response to different intensities and durations of Ca^2+^ signals. The ability of ALG-2 to influence Sec31A targeting may relate to the earlier observation that ALG-2 binding to the Sec31 subunit of the outer coat heterodimer Sec13/31 seems to dramatically potentiate its interaction with the inner coat heterodimer sec23/24^35^; the inner-outer coat interaction is thought to be critical for cargo sorting and vesicle formation.

A completely different line of research has also focused on modification of the COPII subunit Sec31A in response to Ca^2+ ^—and also relates to ALG-2. This work examined the effect of Ca^2+^ dysregulation on the O-GlcNAcylation and membrane targeting of Sec31A^50^. *O*-GlcNAc transferase (OGT) is an enzyme responsible for this newly appreciated cytosolic post-translational modification. OGT was found in a previous article to interact directly with Sec31A and mediate O-GlcNAcylation at position S964^51^. In the more recent article, extremely high Ca^2+^ for 24 hours (brought about with A23187 or A-beta peptide) caused disruption of Sec31A targeting to membranes whereas 24-hour exposure to EGTA caused enhanced Sec31A targeting. The low-Ca^2+^, Sec31A targeting-promoting condition was accompanied by increased O-GlcNACylation of Sec31A and less ALG-2 association whereas the high-Ca^2+^, Sec31A targeting-inhibiting condition was accompanied by decreased O-GlcNACylation and increased ALG-2 association. An S694A mutation of Sec31A that cannot be O-GlcNACylated was not subject to the targeting regulation by Ca^2+^ flux, suggesting that O-GlcNACylation is a powerful determinant of Sec31A targeting. One interesting speculation is that since the ALG-2 binding site on Sec31A (residues 839–851^36^) is relatively close to S694, ALG-2-binding and OGT-binding could be mutually exclusive, in which case Ca^2+^ could regulate Sec31A O-GlcNACylation through Ca^2+^-dependent ALG-2-binding.

The locus of action of Ca^2+^ in the preceding examples is presumed to be the cytoplasm, where released Ca^2+^ regulates the trafficking machinery, such as vesicle coats. However, Ca^2+^ inside the lumen of organelles can also affect trafficking by altering the function of receptors or other vesicle components that influence whether luminal cargo is recruited to a budding vesicle. Substantial depletion of ER Ca^2+^ has been associated with the abnormal secretion of ER-resident proteins normally retained in the ER^52–54^. This abnormal ER exit has been demonstrated to affect proteins containing a C-terminal KDEL retrieval sequence, and their exit can be suppressed by over-expression (OE) of KDEL receptors^55^. Although the mechanism of retrieval via the KDEL receptor is well established, it is not known how luminal Ca^2+ ^contributes to the retention of ER proteins, a phenomenon first noted for the ER chaperone calreticulin in 1994^56^. Several putative mechanisms have been proposed to explain this departure, including (i) proteins with KDEL sequences such as ER chaperones BiP or GRP94 bind Ca^2+^ with low affinity and interact with both each other and misfolded proteins, possibly forming a kind of retention matrix. A decrease in Ca^2+^ could reduce their interactions and cause them to be secreted. Or (ii) the ER environment may exist as a dynamic hydrogel because of a high concentration of Ca^2+^ and Ca^2+^-interacting proteins, and the hydrogel properties may limit access of resident proteins to ERES. A decrease in the Ca^2+^ concentration could thereby decrease the hydrogel viscosity, allowing ER resident proteins to escape and overwhelm the KDEL retrieval pathway. The precise mechanism for Ca^2+^-dependent ER protein retention remains to be explored and will have medical significance to the many diseases whose pathologies involve severe disruption of homeostasis (for example, ischemia)^57^.

The cargo receptor ERGIC-53 (also called LMAN1) is another protein modulated by luminal Ca^2+^. ERGIC-53 Ca^2+^ dependently binds soluble glycoprotein cargoes in the ER lumen^58,59^. Example cargoes include cathepsins C and Z^58,60^, α1-antitrypsin^61^, and serotonin neuroreceptors^62^. In addition, ERGIC-53 forms a 1:1 Ca^2+^-dependent complex with a small Ca^2+^-binding protein MCFD2, both of which are necessary for the trafficking of coagulation factor V (FV) and factor VIII (FVIII)^63^. Mutations in ERGIC-53 or MCFD2 induce the autosomal recessive disorder combined deficiency of factor V and factor VIII (F5F8D)^64^. Previously, ERGIC-53 bound to cargo was thought to assemble in the ER lumen before dissociating in the ERGIC/Golgi in a manner dependent upon Ca^2+ ^release facilitated by decreasing pH between organelle compartments^65^. However, a more recent study found that the carbohydrate-binding domain (CRD) of ERGIC-53 bound to Ca^2+^ was insensitive to the changes in pH predicted to occur between the ER and Golgi^66^. Importantly, that study also showed that CRD-ERGIC-53 had very little binding to a glycan-like moiety below 0.4 mM CaCl_2_. It remains unresolved whether differences in pH between the ER and Golgi, or differences in Ca^2+^ concentration, play a more significant role in regulating ERGIC-53 cargo loading and release.

Other ER luminal proteins also act as cargo receptors. Calumenin (CALU) is a Ca^2+^-binding protein from the CREC (Ca^2+^-binding protein of 45 kDa [Cab45], reticulocalbin, ER Ca^2+^-binding protein of 55 kDa [ERC-55], and calumenin) protein family. In striated muscle, CALU expression changes are coupled to Ca^2+^ cycling likely via Ca^2+^-dependent interaction with the SERCA pump or ryanodine receptors^67^, whereas CALU in thrombocytes may act as a cargo receptor for thrombospondin-1 (TSP1)—an ECM adhesive glycoprotein. In this system, CALU and TSP1 form a Ca^2+^-dependent complex and are released together in response to thrombin^68^. Whether CALU regulates cargo secretion in the same cells in which it regulates ER Ca^2+^ entry/exit remains to be seen. If such a role were observed, this could be an interesting example of a protein that regulates both the Ca^2+^ environment necessary for secretion and the actual secretion event itself.

Highlights of this section are summarized in Figure 2.

### Golgi apparatus

The Golgi apparatus plays a central role in the trafficking and sorting of secretory cargo. Like the ER, the Golgi holds a vast reservoir of Ca^2+^ and is similarly endowed with Ca^2+^ pumps, Ca^2+^ release channels, and Ca^2+^-binding proteins^69^. The Ca^2+^ concentration at the Golgi diminishes from *cis* to *trans* (300 –> 150 μM), reaching the lowest concentration at the trans-Golgi network (TGN) (50–100 μM)^9^ (as depicted in Figure 1). Maintenance of this Ca^2+^ gradient appears to require a gradient of protein expression. Notably, the cis-Golgi maintains SERCA pumps for Ca^2+^ influx and IP3R channels for Ca^2+^ release, whereas, in the trans-Golgi compartment, Ca^2+^ transport is regulated mainly by the secretory pathway Ca^2+^ ATPase type 1 (SPCA1) and ryanodine receptors^9,69–71^. Similarly, three Golgi proteins considered Ca^2+^ regulators, two of which have direct roles in secretion (discussed below), are localized to distinct Golgi compartments: nucleobindin-1 in the cis-Golgi^72–74^, DNA-binding EF-hand acidic amino acid-rich non-glycosylated Ca^2+^-binding protein (NEFA/p54) in the medial-Golgi^75^, and Cab45 in the trans-Golgi and TGN^76,77^. It is possible that just as ERGIC-53 may use Ca^2+^ to regulate cargo loading and release, distinctive Ca^2+^ levels in the Golgi could aid cargo progression (although this is not directly explored here). In this section, we define specific instances of Ca^2+^ regulating secretion via its flux near the cytosolic surface or within luminal Golgi compartments.

Many studies have implicated Golgi Ca^2+^ channels and Ca^2+^ effectors in regulating trafficking. To begin, we discuss Sly41, an SLC-family solute transporter that constitutively cycles between the ER and Golgi in *Saccharomyces cerevisiae*. Although the substrate for the transporter remains unknown, genetic interactions with the PMR1 Golgi Ca^2+^ pump led to the discovery that Sly41 OE generated elevated cytosolic Ca^2+^^78^. This was an important trafficking discovery since Sly41 was originally identified in a screen for genes whose OE suppressed COPII-dependent membrane tethering deficiencies (SLY = suppressor of lethality of ypt1). Mechanistically, this meant that raising the cytosolic Ca^2+^ concentration through Sly41 OE compensated for the loss of an ER-to-Golgi vesicle tethering factor (Uso1; p115 in mammals) that is essential for intracellular transport pathways and efficient SNARE assembly^78,79^. These findings, on their own, suggest that Ca^2+^ promotes membrane fusion between COPII vesicles and the Golgi. Curiously, although the addition of Ca^2+ ^in the absence of maximal vesicle tethering factors did indeed stimulate membrane fusion, it did nothing when saturating levels of vesicle tethering factors were present. A Ca^2+^ effector protein such as ALG-2 or calmodulin (CaM) that, for example, interacts with SNAREs or tethers and mediates the stimulation of transport was not identified. However, some Ca^2+^ effects on membrane fusion could instead be due to the fusion-promoting properties of Ca^2+^ itself without the requirement for a Ca^2+^ effector (to be discussed below).

A recent study investigated the effect of high cytosolic Ca^2+^ concentrations on Golgi structure^80^. The authors exposed HeLa cells to the Ca^2+^ agonist histamine or the SERCA inhibitor thapsigargin for 0.5 to 2 hours. They noted morphological fragmentation of Golgi stacks via a mechanism that involved PKCα-mediated phosphorylation of Golgi reassembly stacking protein 55 (GRASP-55). The fragmentation response appeared to be independent of ER stress and UPR activation. Satisfyingly, in this example, we have both an established Ca^2+^ effector (PKCα) and the trafficking machinery impacted (GRASP-55). However, the trafficking purpose for the effect is less clear. The authors suggested that it was part of a Golgi stress adaptive mechanism or signaling pathway. Cargo trafficking effects through the Golgi were regarded as mild; however, there was measurable acceleration that could have contributed to increased secretion. It would be interesting to know whether the PKCα-mediated response to Ca^2+^ signaling in the Golgi works in concert with changes in ER export mediated by ALG-2 that were discussed above.

As with the ER possessing TRPP2, the Golgi also possesses resident TRP channels that appear to affect secretion. The transient receptor potential canonical (TRPC) sub-family of TRP channels are non-selective cation channels that are known to open in response to phospholipase C activation, although their precise ligands (IP3, diacylglycerol) vary^81^. Human TRPC3 and 7 are localized on the PM but also are found in intracellular pools at the TGN and Golgi stack^82^. Furthermore, OE of either of these channels was found to increase constitutive secretion of SEAP by two- to four-fold^82^. The precise locus of the secretion effects observed in that study was not elucidated, and it cannot be eliminated that the over-expressed channels mobilized ER Ca^2+^ in addition to Golgi Ca^2+^ and that ER Ca^2+^ was the functional site, possibly via the effects of ALG-2 on COPII targeting, as discussed above. However, it is tempting to speculate that what occurred was local stimulation of intra-Golgi cargo transport, a process conventionally thought to be facilitated, through the process of cisternal maturation, by COPI-coated vesicles (although a competing hypothesis is intra-Golgi tubules, discussed below). Interestingly, two groups have presented evidence that COPI targeting to membranes, like COPII targeting, is Ca^2+^-dependent^34,83^ but that ALG-2 did not affect COPI targeting^34^. It would be very exciting if a separate Ca^2+^ effector regulated COPI; furthermore, this putative effector could be the key to understanding TRPC effects on secretion.

A prominent Golgi Ca^2+^ effector is the cytosolic phospholipase A_2_ alpha (cPLA_2_α). cPLA_2_α is a member of the superfamily of phospholipase A_2_ enzymes (PLA_2_) reviewed extensively elsewhere^84^. In general, PLA_2_ hydrolyze the fatty acid from the sn-2 position of membrane phospholipids creating a lysophospholipid and a free fatty acid. An inverted-cone-shaped lipid, the lysophospholipid favors positive curvature of lipid membranes and thereby promotes membrane tubule formation. Tubule-mediated (as opposed to vesicle-mediated) trafficking is gaining recognition for its contributions to intra-Golgi transport as well as Golgi-to-PM transport of certain cargoes^85^. Three specific PLA_2_ enzymes have been implicated in tubule regulation at the Golgi^85–87^, including cPLA_2_α^88^. Whereas cPLA_2_α is Ca^2+^ activated, the other two Golgi-associated PLAs are not but could instead be activated by G protein βγ complexes^89^. Notably, cPLA_2_α is specifically recruited to the Golgi complex, likely via its C2 domain^90^, in response to transient increases in Ca^2+^ apparently initiated by the arrival of secretory cargo^88^. There it was required for intra-Golgi transport of VSV-G through the Golgi, apparently via induction of small inter-stack tubules. In the study by San Pietro *et al*., depletion of cPLA_2_α specifically inhibited transport through the Golgi and did not impair ER-to-Golgi transport of VSV-G or delivery of VSV-G from the TGN to the PM or retrograde Golgi-to-ER transport of the KDEL receptor^88^. Another article showed that cPLA_2_α contributes to the delivery of transmembrane proteins to junction complexes in endothelial cells^91^. This point was demonstrated by noting that the inhibition or RNA interference-mediated KD of cPLA_2_α prevented delivery of VE-cadherin, occludin, and claudin-5 to cell-to-cell contacts. Instead, these proteins accumulated in the Golgi, indicating that either transport through the Golgi or transport from it was inhibited^91^. The evidence for cPLA_2_α in the regulation of intra-Golgi transport is thus quite strong, but the Ca^2+^ signaling that activates cPLA_2_α on the Golgi is less clear. One report suggested that Ca^2+^ is released from the Golgi itself^92^ when a bolus of secretory cargo enters the Golgi, although specific channels or pumps and their mode of activation were not identified.

Secretory pathway Ca^2+^-ATPase (SPCA1) is a primarily trans*-*Golgi-localized Ca^2+^ ATPase^69,70^, which, along with the ER SERCA pump, is responsible for the maintenance of Golgi Ca^2+^ levels. Mutations in SPCA1 (gene name: *ATP2C1*) usually manifest as a decrease in expression and elicit the autosomal dominant skin disorder Hailey-Hailey disease^93^. As we will see in the following examples, the role of SPCA1 in secretion seems to be mainly in providing the lumen of the Golgi and associated organelles with high Ca^2+^ that is used in sorting and processing of secretory cargoes en route to their final destinations. The role of SPCA1 in trafficking was first demonstrated in yeast, where its medial Golgi-localized ortholog PMR1 transports both Ca^2+^ and Mn^2+^ into secretory compartments^94^. In that early study, *pmr1* mutants are both unable to sort carboxypeptidase properly to the vacuole and unable to degrade misfolded carboxypeptidase, a luminal ER protein—a trait that was later found to be reversible via addition of external calcium^95^. This suggested that low luminal calcium in secretory organelles is the primary cause of these sorting defects. Further studies reinforce this idea. For example, SPCA1 inhibition also produced a decrease in insulin-like growth factor receptor (IGF1R) at the cell surface, a phenotype caused by defective proteolytic processing and accumulation of previously undetectable TGN-localized pro-IGF1R^96^. One putative effector for the sorting role of SPCA1 in secretion arose later when it was discovered that the luminal, secreted Ca^2+^-binding protein Cab45, of the CREC family, preferentially accumulates near SPCA1, where it oligomerizes with secretory cargoes in response to the presumably high local concentration of Ca^2+^^97^. Cab45 does not play a general role in secretion but rather binds a few select secretory cargoes, including lysozyme C and cartilage oligomeric matrix protein (COMP), and presumably assists their secretion by forming condensates^97,98^. Curiously, Cab45 is secreted along with its client cargoes. This could imply that Cab45 has additional functions in the extracellular environment.

Nucleobindin-1 (NUCB1, also called CALNUC or NUC) appears to be a multi-functional soluble EF-hand-containing Ca^2+^ effector protein. NUCB1 is widely distributed within the cell. The protein contains both an ER signal sequence and an ER export signal and displays prominent localization to the cis Golgi^72–74^ prior to being constitutively secreted^99^. Surprisingly, NUCB1 is also present in the cytoplasm^100^. In the cytoplasm, it has been suggested to, among other things, undergo Ca^2+^-dependent interactions with G_⍺_i subunits on Golgi membranes^101,102^. Importantly, cytosolic NUCB1 acts as a regulator of endosomal recycling of lysosomal receptors such as the mannose-6-phosphate receptor and sortilin, which capture cargo at the TGN and ferry it to late endosomes for ultimate deposition in the lysosome^100,103^. In these studies, NUCB1 appeared to act in the cytosol and as a regulator of rab7 activation to bring about recruitment of the retrograde coat, retromer, that mediates return of the lysosomal receptors to the TGN. The role of Ca^2+^ in NUCB1 function here was not investigated.

In regard to NUCB1 that is targeted to the luminal cellular domain, some reports have suggested a chaperone-like activity due to its ability to bind Alzheimer’s amyloid precursor protein (APP) and assist in its folding and biogenesis^104,105^. In the Golgi, NUCB1 may function as a regulator of Golgi Ca^2+^ homeostasis since its OE increased Ca^2+^ uptake into the Golgi^73^ while NUCB1 KO induced a loss in Golgi luminal Ca^2+^^106^. Furthermore, NUCB1 KO and NUCB1 with mutant EF hands produced a delay in the trafficking of ECM constituents MMP2 and MT1-MMP at the cis-to-trans Golgi stage. Again, like Cab45, NUCB1 appeared to regulate the trafficking of only a subset of cargoes; for example, NUCB1 KO had no effect on the trafficking of lysozyme C^106^. This trafficking defect was a delay and not a block, possibly due to compensatory mechanisms mediated by other Ca^2+^-binding proteins. The trafficking function of NUCB1 was presumed to be mediated by direct interactions with the cargo, but a more detailed mechanism of how NUC1B facilitates anterograde transport while remaining in the cis-Golgi has not yet been established. Finally, NUCB1 is reported to, after a long period in the Golgi, be constitutively secreted into the extracellular space^99^, where it may have signaling functions via its capacity to bind Ca^2+^, such as in bone matrix maturation^107^.

Highlights of this section are summarized in Figure 3.

### Late endosome/lysosome

The late endosomes/lysosomes (LELs) are central organelles responsible for macromolecule recycling. Importantly, they are also intracellular stores for Ca^2+^, reaching concentrations that approach that of the ER lumen^108^. We have known for quite some time that LEL trafficking requires luminal Ca^2+^ and that CaM is an important Ca^2+^ effector for this^5,6,109^. Resident in the lysosome is the TRPML1 channel that is critical in Ca^2+^-dependent lysosome trafficking; lack of TRPML1 activity causes the genetic disorder mucolipidosis and enlarged vacuole-like lysosomes^110^. TRPML1 belongs to the mucolipin subgroup of the TRP ion channel family whose members we have discussed in the sections on trafficking in the ER and the Golgi. Like other TRP channels, TRPML1 acts as a non-selective ion channel permeable to Ca^2+^. Some work has indicated that TRPML1 is an activator of lysosome-MVB fusion. In this context, TRPML1 was suggested to regulate exosome release since this process is inhibited when lysosomes fuse with MVBs, the source of exosomes^111,112^. Other work, however, has implicated TRPML1 in LEL fission^113^. TRPML1 has been shown to be regulated by pH^108^ and activated by the rare LEL phospholipid PI(3,5)P_2_. Deficiency of either TRPML1 or PI(3,5)P_2_ produced enlarged LEL, while OE of TRMPL1 both attenuated the defect observed with PI(3,5)P_2_ deficiency and enhanced vacuolar calcium release^114^. This local increase in juxta-organellar calcium could serve to recruit cytosolic complexes necessary for membrane fusion and fission. In fact, CaM was implicated as the specific Ca^2+^ effector for the role of TRPML1 in LEL fission^113^, and the mammalian target of rapamycin 1 (mTORC1) served as a required downstream target^115^. ALG-2 (discussed above in the ER section) has also been implicated as an effector of Ca^2+^ released by TRPML1 since it binds to TRPML1 in a Ca^2+^-dependent manner^116^. Furthermore, activation of TRPML1 via PI(3,5)P_2_ caused ALG-2 to Ca^2+^-dependently couple dynein-dynactin with the N-terminus of TRPML1^10^. This ALG-2-mediated adaptor system ultimately permitted migration of lysosomes to the perinuclear region in response to Ca^2+^ signals. Distinct from the proposed role of CaM in LEL fission, this dynein-mediated trafficking implicates Ca^2+^ and ALG-2 in autolysosome formation, which occurs when the migrated lysosomes fuse with autophagosomes.

Also localized to LEL, purinergic receptor P2X4 is a Ca^2+^ release channel activated by luminal ATP in a pH-dependent manner. In a 2015 article, it was shown that P2X4 promotes LEL fusion in a cell-free assay and that this effect was blocked by various inhibitions of P2X4-mediated Ca^2+^ release^117^. Although inhibition of P2X4 activity by the low pH of LEL was overcome pharmacologically in these experiments, it is unknown whether and how this inhibition is overcome in cells. That study also showed that P2X4 activation recruits CaM and that together they form a complex at the LEL membrane. Finally, the fusion effect of P2X4 was shown to be suppressed by inhibiting CaM^117^. The authors do not specifically identify the downstream targets for CaM in this instance, although it may be relevant that CaM interacts with LEL SNARE proteins in a Ca^2+^-dependent manner^118^. It is interesting to note that the same research group also implicated CaM as the effector for TRPML1-mediated LEL fission^113^, but how two distinct channels could mediate opposing trafficking phenomena (fusion vs. fission) via the same Ca^2+^ effector was not resolved.

Highlights of this section are summarized in Figure 4.

### Baseline exocytosis of secretory granules

Specialized epithelial goblet cells secrete gel-forming mucins, the first line of defense against pathogens or allergens. Following biogenesis at the Golgi, mucin granules undergo maturation and eventually release their contents at the PM. Although mucin granules undergo stimulated exocytosis in response to agonists such as ATP, it was discovered in 2015 that baseline secretion of mucins, which requires intracellular Ca^2+^ concentrations an order of magnitude lower than agonist-stimulated release^119^, can exceed stimulated release over long periods^120^. A high-affinity Ca^2+^ sensor for baseline mucin secretion is proposed to be K^+^ channel-interacting protein 3 (KChIP3), a member of the neuronal Ca^2+ ^sensor (NCS) family of EF-hand-containing proteins^121^. KChIP3 inhibits baseline exocytosis, which was shown by an increase in baseline mucin secretion during KChIP3 KD and a decrease in baseline secretion during KChIP3 OE. This effect was demonstrated to be entirely dependent on intracellular Ca^2+^ oscillations, as increased intracellular calcium oscillations abrogated the inhibitory effect of KChIP3 OE. Finally, this Ca^2+^-dependent control of KChIP3 activity was discovered to be dependent on the ryanodine receptors, activity of which caused KChIP3 to dissociate from mucin granules^121^. Although this process sounds more akin to regulated secretion, it was able to proceed without stimulation by external agonists and did not require synaptotagmin, thus blurring the distinction between the regulated and constitutive secretory pathways. *In vivo*, the Ca^2+^ oscillations may be driven by fluid flow over the epithelium, as goblet cells were found to increase Ca^2+^ oscillations and mucin secretion in response to cell perfusion^120^.

## Calcium and membranes

Secretory pathway organelles harbor significant stores of calcium. The ER is reported to be the highest with a total Ca^2+^ of up to 2 mM, equivalent to the extracellular Ca^2+^ concentration, whereas its free Ca^2+^ store is about 500 μM^8^. Meanwhile, the Golgi apparatus sits at an average of 250 μM free Ca^2+^^8^, although this number varies; Ca^2+ ^steadily diminishes from the cis- to the trans-Golgi compartments^122^. Finally, the average free Ca^2+^ of lysosomes is comparable to the ER at about 500 μM^8,10^. Although so far we have discussed the influence of these stores primarily in the context of their capacity to regulate Ca^2+^-binding proteins that in turn regulate vesicle trafficking, here we discuss the possibility that Ca^2+^ directly modulates trafficking by influencing the structural and functional properties of membrane lipids. Indeed, experimental and theoretical work has shown that Ca^2+^ can quickly and concentration-dependently tighten and order lipid bilayers, primarily via coordination with anionic oxygens of acidic phospholipids such as phosphatidylinositol-4,5-bisphosphate [PI(4,5)P_2_]^123,124^ and phosphatidylserine (PS)^125–127^. Together these studies suggested a role for cytosolic Ca^2+^ in promoting membrane fusion, possibly by masking the negative charge of anionic lipids and more readily allowing close association of membranes. Ca^2+^ concentrations on the order of 200 to 400 μM overcame DOPS (dioleoyl phosphatidylserine) inhibition of lipid or content mixing in prepared vesicles^128^. Importantly, this Ca^2+^ concentration is on the order one might expect for focal Ca^2+^ signals near release sites^129,130^. We note that, in a previously discussed study, elevations in cytosolic Ca^2+ ^were observed to overcome a block in membrane tethering to allow fusion^78^. Since no Ca^2+^ effector was evident in that study, it could indicate that Ca^2+^ alone may have promoted membrane fusion in a physiological system.

In addition to putative direct effects on fusion, Ca^2+^ may have additional roles in vesicular trafficking. Studies have shown, for example, induction of membrane tubulation in lipid vesicles in response to local Ca^2+^ addition at concentrations above 1 mM^131^. The authors of that study note that the observed inward spontaneous curvature and membrane bending are likely due to a large, Ca^2+^-facilitated reduction in the surface charge density of one membrane leaflet causing stress asymmetry and ultimately bending of the membrane. A study using a giant unilamellar vesicle transfer assay demonstrated that Ca^2+^ can trigger deformation of membranes containing PS or PI(4,5)P_2_ in a direction that points away from the ion source^132^, in a manner reminiscent of vesicle budding. It is important to note that the concentration of Ca^2+^ required to manifest the induced curvature of membranes appears to be on the order of a few hundred micromolar. Although this number falls well within the bounds of free luminal Ca^2+^ for secretory pathway organelles, it is highly unlikely that high levels of free Ca^2+^ alone could lead to a membrane budding event. Instead, it is much more plausible that luminal Ca^2+^ stores support induction of membrane curvature by working in concert with other proteins such as the annexins^133^. Finally, in support of a system wherein Ca^2+^ can facilitate membrane budding, we note that the membranes of organelles most enriched in the Ca^2+^-binding lipid PS are the luminal leaflets of the ER, Golgi, and mitochondria^134,135^, two of which are critical organelles for constitutive secretion.

## Conclusions

Since our last review of this topic^7^, Ca^2+^ regulation of constitutive trafficking has expanded from examples employing mostly *in vitro* systems using Ca^2+^ addition or chelation into complex systems wherein specific Ca^2+^-related gene products, many implicated in human pathologies, fundamentally alter trafficking steps throughout secretory and LEL trafficking. Furthermore, whereas prior to this it was difficult to distinguish whether Ca^2+^ played only permissive, co-factor roles, now we know that Ca^2+^ signaling can play dynamic roles, altering trafficking rates or patterns in response to natural ligands. Furthermore, we note that Ca^2+^ signaling can have unexpectedly complex roles; for example, in the case of ER export, ALG-2, together with its multiple targets, can act to either restrict or expand entry of cargo into the secretory pathway, depending upon the intensity and duration of a Ca^2+^ signal. It is now clear that Ca^2+^ is a fundamental regulator of the cell’s trafficking toolkit but that each case is also mechanistically unique rather than following a universal pattern. Overall, constitutive trafficking is looking a lot more Ca^2+^-regulated and therefore less “constitutive” than once believed.
